# Functional foods and dietary supplements in the management of non-alcoholic fatty liver disease: A systematic review and meta-analysis

**DOI:** 10.3389/fnut.2023.1014010

**Published:** 2023-02-14

**Authors:** Lei-lei Wang, Pian-hong Zhang, Hui-hui Yan

**Affiliations:** ^1^Department of Clinical Nutrition, The Second Affiliated Hospital, College of Medicine, Zhejiang University, Hangzhou, Zhejiang, China; ^2^Department of Gastroenterology, The Second Affiliated Hospital, College of Medicine, Zhejiang University, Hangzhou, Zhejiang, China

**Keywords:** non-alcoholic fatty liver disease, antioxidants, phytonutrients, probiotics, symbiotics, prebiotics

## Abstract

**Objective:**

In this systematic review and meta-analysis, we aimed to clarify the overall effects of functional foods and dietary supplements in non-alcoholic fatty liver disease (NAFLD) patients.

**Methods:**

Randomized controlled trials (RCTs) published in PubMed, ISI Web of Science, Cochrane library, and Embase from January 1, 2000 to January 31, 2022 were systematically searched to assess the effects of functional foods and dietary supplements in patients with NAFLD. The primary outcomes were liver-related measures, such as alanine aminotransferase (ALT), aspartate aminotransferase (AST), and hepatic fibrosis and steatosis, while the secondary outcomes included body mass index (BMI), waist circumference (WC), triacylglyceride (TG), total cholesterol (TC), low-density lipoprotein cholesterol (LDL-C), and high-density lipoprotein cholesterol (HDL-C). These indexes were all continuous variables, so the mean difference (MD) was used for calculating the effect size. Random-effects or fixed-effects models were used to estimate the mean difference (MD). The risk of bias in all studies was assessed with guidance provided in the Cochrane Handbook for Systematic Reviews of Interventions.

**Results:**

Twenty-nine articles investigating functional foods and dietary supplements [antioxidants (phytonutrients and coenzyme Q10) = 18, probiotics/symbiotic/prebiotic = 6, fatty acids = 3, vitamin D = 1, and whole grain = 1] met the eligibility criteria. Our results showed that antioxidants could significantly reduce WC (MD: −1.28 cm; 95% CI: −1.58, −0.99, *P* < 0.05), ALT (MD: −7.65 IU/L; 95% CI: −11.14, −4.16, *P* < 0.001), AST (MD: −4.26 IU/L; 95% CI: −5.76, −2.76, *P* < 0.001), and LDL-C (MD: −0.24 mg/dL; 95% CI: −0.46, −0.02, *P* < 0.05) increased in patients with NAFLD but had no effect on BMI, TG, and TC. Probiotic/symbiotic/prebiotic supplementation could decrease BMI (MD: −0.57 kg/m^2^; 95% CI: −0.72, −0.42, *P* < 0.05), ALT (MD: −3.96 IU/L; 95% CI: −5.24, −2.69, *P* < 0.001), and AST (MD: −2.76; 95% CI: −3.97, −1.56, *P* < 0.0001) levels but did not have beneficial effects on serum lipid levels compared to the control group. Moreover, the efficacy of fatty acids for treating NAFLD was full of discrepancies. Additionally, vitamin D had no significant effect on BMI, liver transaminase, and serum lipids, while whole grain could reduce ALT and AST but did not affect serum lipid levels.

**Conclusion:**

The current study suggests that antioxidant and probiotic/symbiotic/prebiotic supplements may be a promising regimen for NAFLD patients. However, the usage of fatty acids, vitamin D, and whole grain in clinical treatment is uncertain. Further exploration of the efficacy ranks of functional foods and dietary supplements is needed to provide a reliable basis for clinical application.

**Systematic review registration:**

https://www.crd.york.ac.uk/prospero, identifier: CRD42022351763.

## 1. Introduction

Non-alcoholic fatty liver disease (NAFLD), renamed metabolic-associated fatty liver disease (MAFLD) in 2020 (1), is a chronic and progressive metabolic disease characterized by excessive fat deposition in the hepatocytes in the absence of alcohol exposure or other identifiable causes. This may finally lead to cirrhosis, hepatocellular carcinoma (HCC), and other severe liver diseases (2, 3), adversely impacting people's quality of life. It is reported that the global prevalence of NAFLD is 24.1%, of which the highest are in the Middle East and South America and the lowest are in Africa (4, 5). The prevalence of NAFLD in China is about 30%, and this rate is expected to increase further as obesity and type 2 diabetes mellitus (T2DM) are gradually becoming the most common diseases in humans (6, 7).

The occurrence and development of NAFLD depend on multiple factors, including metabolic comorbidities, gut microbiome, and environmental and genetic factors (8). Except for the genetic factors that human beings cannot intervene, the other three conditions are closely related to our current lifestyle and affect each other. In particular, they are influenced by a sedentary lifestyle and the intake of high-energy foods, including fried foods, desserts, and soft drinks (9). The habits mentioned above account for obesity, T2DM, and hyperlipidemia, which are major risk factors for NAFLD (10). It is reported that the prevalence of NAFLD is as high as 60–90%, 27–92%, and 28–70% in patients with obesity, hyperlipidemia, and T2DM, respectively (6).

As no approved pharmacological options exist for NAFLD, lifestyle modifications, including increased physical activity and reduced energy intake, remain the first line of therapy (10, 11); however, consistent low-energy intake and exercise are difficult to achieve in both adults and children. Recently, researchers have found that gut microbiota plays an important role in the pathophysiology of metabolic diseases, especially obesity-related disorders, including metabolic syndrome and NAFLD (12). The hypothesis underlying its mechanism is that decreased microbial gene richness leads to a decline in bacteria producing short-chain fatty acids and an increase in bacteria synthesizing lipopolysaccharide, which can induce the development of steatosis and trigger systemic inflammation (13, 14). Early probiotic, prebiotic, symbiotic, and fecal microbiota transfer interventions may prevent NAFLD exacerbation (15, 16). Meanwhile, some investigations have reported that the intake of dietary supplements (such as vitamin D, vitamin E, and fatty acid) (17, 18) and functional foods (such as garlic powder, *Nigella sativa*, sumac powder, and olive oil) (19–23) may ameliorate and/or reverse NAFLD, which may be due to their antioxidant properties. For example, organosulfur compounds in garlic powder and flavonoids in green tea extract belong to the category of functional foods, but in terms of properties, they exhibit antioxidant activities. Their protection is mainly achieved in NAFLD by decreasing oxidative stress and inflammatory responses (12, 20, 24). Although some functional foods may have other properties, such as anticancer and antimicrobial effects (12, 25), these effects on NAFLD are unclear and need to be further explored.

Some functional foods and dietary supplements may decrease liver damage, such as liver disease without cirrhosis, there are inconsistencies in the results obtained by different studies. Considering the inconsistencies of clinical randomized controlled trials (RCTs), we performed this systematic review and meta-analysis to provide clinicians, nutritionists, and other health professionals with relatively unified clinical evidence to help them make relevant clinical decisions, that is, whether functional foods and dietary supplements should be applied to NAFLD management.

## 2. Materials and methods

### 2.1. Eligibility criteria

This study was only limited to RCTs that reported the effects of functional foods and dietary supplements on liver-related indices in patients suffering from NAFLD, regardless of the method of diagnosis [fibroscan/ultrasonography/elastography technique/magnetic resonance spectroscopy/acoustic structure quantification (ASQ) liver scan/percutaneous liver biopsy/computed tomography (CT)], obesity status, and other metabolic syndrome statuses. Animal or *in vitro* studies, case reports, abstracts, guidelines, reviews, meta-analyses, and conference proceedings were excluded. Two reviewers determined the inclusion and exclusion criteria jointly and retrieved articles separately.

#### 2.1.1. Inclusion criteria

(1) Patients were adults (≥18-year-old) with NAFLD; (2) interventions in studies included: dietary supplementation (synbiotic, symbiotic, prebiotic, probiotic, vitamin, and omega-3 supplementation) and functional foods only (exhibiting effects, including antioxidant, anti-inflammatory, immunomodulatory, antimicrobial action); (3) the placebo in the control group was identical in color, shape, size, and packaging to that of the intervention group; (4) the main outcomes were the changes in alanine aminotransferase (ALT) and/or aspartate aminotransferase (AST), hepatic fibrosis, and hepatic steatosis; (5) there were no restrictions on the duration of intervention; (6) only publications written in English were chosen, but the country was not a limiting factor.

#### 2.1.2. Exclusion criteria

(1) Patients with advanced liver disease or/and gastrointestinal dysfunction; (2) interventions in studies combined with exercise or/and active lifestyle modification, medicine, weight loss surgery, and enteral and/or parenteral nutrition treatment; (3) studies that did not report transaminase values (AST and/or ALT).

### 2.2. Search strategy

This systematic review and meta-analysis was conducted according to the Preferred Reporting Items for Systematic Reviews and Meta-Analyses Statement (PRISMA). Its protocol was registered on PROSPERO (CRD42022351763). One of the researchers was responsible for keyword searches across four major electronic databases: PubMed, ISI Web of Science, Cochrane library, and Embase, from January 1, 2000 to January 31, 2022. The search strategy and results were finalized through group discussions.

The search method was as follows: (“non-alcoholic fatty liver disease” OR “NAFLD” OR “nonalcoholic fatty liver disease” OR “fatty liver, nonalcoholic” OR “fatty livers, nonalcoholic” OR “liver, nonalcoholic fatty” OR “livers, nonalcoholic fatty” OR “nonalcoholic fatty liver” OR “nonalcoholic fatty livers” OR “nonalcoholic steatohepatitis” OR “nonalcoholic steatohepatitides” OR “steatohepatitides, nonalcoholic” OR “steatohepatitis, nonalcoholic”) AND (“diet” OR “diets” OR “dietary supplements” OR “dietary supplement” OR “supplements, dietary” OR “dietary supplementations” OR “supplementations, dietary” OR “food supplementations” OR “food supplements” OR “food supplement” OR “supplement, food” OR “supplements, food” OR “nutraceuticals” OR “nutraceutical” OR “nutriceuticals” OR “nutriceutical” OR “neutraceuticals” OR “neutraceutical” OR “herbal supplements” OR “herbal supplement” OR “supplement, herbal” OR “supplements, herbal” OR “nutrients” OR “nutrient” OR “macronutrients” OR “macronutrient” OR “micronutrient” OR “micronutrients” OR “functional food” OR “food, functional” OR “foods, functional” OR “functional foods”) AND (“randomized controlled trial” [publication type] OR “randomized controlled trial” OR “randomized” OR “placebo”).

**Figure 1:**
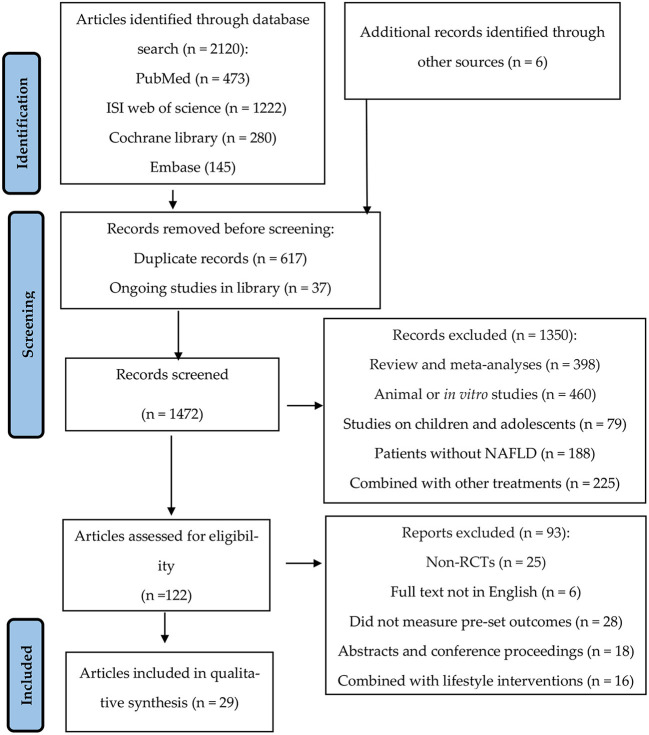
Preferred reporting items for systematic reviews and meta-analyses (PRISMA) flow diagram detailing the study selection process for systematic literature review.

Literature was managed using a software program (EndNote X8.1, Thomson Reuters, New York, USA), in which the “find duplicates” function was used to eliminate duplicate papers. The studies inconsistent with our eligibility criteria were screened out using the title and abstract. The remaining articles were fully read, and the qualified studies were chosen according to the inclusion and exclusion criteria (Figure 1). All stages of the selection process were repeated by another author, and the differences were solved through group discussion.

### 2.3. Data extraction and risk of bias assessment

The data from each trial were extracted independently by two authors using a standardized, pre-designed data-extraction form. The data included: (1) study characteristics (first author's name, year of publication, country, and sample size); (2) baseline patient characteristics (targeted population and age); (3) intervention characteristics (type and dose in the intervention and control groups and duration of follow-up); (4) main outcomes (outcome measures and the changes to outcome measures post-intervention). The risk of bias in all studies was assessed using the Cochrane Risk of Bias Tool (RoB2) provided in the Cochrane Handbook for Systematic Reviews of Interventions (26). The tool contains seven domains, including method of sequence generation, concealment, blinding, blinded outcome, incomplete outcome data, selective outcome reporting, and other biases. All these domains were respectively classified as low risk, high risk, and unclear risk of bias according to the guideline in the Cochrane Handbook. The trial was considered to have “good” quality if it was low-risk for at least three items, “fair” if it was low-risk for two items, and “weak” if it was low-risk for less than two items (27, 28). The quality assessment was separately done by two authors. There was little disagreement between them.

### 2.4. Outcome measures

The primary outcomes were liver-related measures, such as transaminase values (AST and/or ALT), and hepatic fibrosis and steatosis. Secondary outcomes included anthropometric measurements [such as body mass index (BMI) and waist circumference (WC)] and lipid analyses [triacylglyceride (TG), total cholesterol (TC), low-density lipoprotein cholesterol (LDL-C), and high-density lipoprotein cholesterol (HDL-C)].

### 2.5. Statistical analysis

The mean differences (MD) and 95% confidence interval (CI) between intervention and placebo groups at baseline and post-intervention were used for each parameter in this review and meta-analysis. *I*^2^ value was used to evaluate heterogeneity. When the *I*^2^ value was < 50%, the fixed-effects model was used; otherwise, the random-effects model was adopted. We used a narrative synthesis when the data were too heterogeneous to be aggregated or when there were only a few articles on intervention measures (29). The publication bias was evaluated using Begg's rank correlation test and Egger's regression asymmetry test. Sensitivity analysis was carried out to define whether the overall effect depended on any particular study. The statistical significance was assessed at α = 0.05. All statistical analyses were carried out using Review Manager 5.4 and Stata 12.0.

## 3. Results

### 3.1. Study selection

The study selection process is summarized in Figure 1. We selected 2,120 articles through keywords and six articles through references, from which 617 duplicate and 37 ongoing studies were removed. Next, we excluded 1,350 studies after screening the title and abstract. A total of 122 studies were considered eligible and needed a full-text review. Finally, 93 articles were excluded after reading the full text because of the following reasons: non-RCTs; full text not in English; did not measure pre-set outcomes (AST/ALT/hepatic fibrosis and steatosis); abstracts and conference proceedings; and combined with lifestyle interventions (physical exercise and/or weight loss diet). Finally, 29 articles were included in this review and meta-analysis.

### 3.2. Study characteristics

The characteristics of included studies are summarized in Table 1. Fifteen trials (20, 21, 23, 30–41) evaluated phytonutrients; six trials (42–47) evaluated probiotic/symbiotic/prebiotic; three trials (48–50) evaluated coenzyme Q10; three trials (51–53) evaluated fatty acids; and one trial (54, 55) evaluated vitamin D and whole grain separately in the management of NAFLD. The duration of the intervention varied from 4 weeks to 18 months. The patients were all adults, and the studies were from different countries, including Iran, Korea, Brazil, North America, the United Kingdom, Italy, Malaysia, and China. Among the 29 included studies, 23 (79.31%) articles studied patients with NAFLD only, 2 (6.90%) articles studied NAFLD patients with diabetes, and 4 (13.79%) articles were on overweight/obese patients with NAFLD. The study design of all included RCTs was parallel (Table 1).

**Table 1:** Characteristics of included trials.

**References**	**Country**	**Sample size**	**Target population**	**Age (years)**	**Duration**	**Intervention group**	**Control group**	**Outcomes measures**	**Changes to outcome measures post intervention**
Aller et al. (42)	Spain	30	NAFLD	≥18	3 months	*Lactobacillus bulgaricus* and *Streptococcus thermophilus*	Starch	BMI/weight/FM/WHR/TC/TG/LDL/HDL/insulin/ HOMA/TNF-α/ALT/AST/GGT	↓ALT/AST/GGT
Sangouni et al. (23)	Iran	90	NAFLD	≥18	12 weeks	Garlic powder	Starch	Hepatic steatosis/weight/BMI/WC/ALT/ASL/GGT/ALP/ TC/TAG/HDL/LDL	↓Hepatic steatosis/ALT/ASL/GGT/TC/HDL/LDL
Pezeshki et al. (30)	Iran	80	NAFLD with obesity	20–50	12 weeks	Green tea extract	Placebo	Weight/BMI/ALT/AST/ALP	↓ALT/AST/ALP
Asgharian et al. (43)	Iran	80	NAFLD	18–60	8 weeks	Probiotic	Starch	Steatosis grade/ALT/AST/CRP/weight/BMI	↓Steatosis grade
Han et al. (31)	Korea	96	NAFLD	≥18	8 weeks	SPB-201	Crystallin cellulose	ALT/AST/blood RT/ALP/glucose/BUN/Cr/UA	↓ALT/AST
Soleimani et al. (garlic) (20)	Iran	110	NAFLD	20–70	15 weeks	Garlic powder	Placebo	Weight/TC/TG/LDL/HDL/FBS/HbA1c/ALT/AST/ hepatic steatosis	↓Hepatic steatosis/weight/TC/TG/LDL/FBS/HbA1c/ ALT/AST
Soleimani et al. (Propolis) (32)	Iran	54	NAFLD	18–60	4 months	Poplar propolis	Placebo	Weight/fat mass/fat free mass/FBS/insulin/HOMA-IR/QUICKI/TC/TG/LDL/HDL/Hs-CRP/albumin/ALP/ALT/AST/GGT/T-Bil/D-Bil/hepatic steatosis	↓Hs-CRP//hepatic steatosis
Scorletti et al. (DHA) (53)	UK	103	NAFLD	≥18	15 months	DHA/EPA	Olive oil	BMI/WC/FBS/MRI subcutaneous fat/MRI visceral fat/HbA1c/TG/TC/LDL/HDL/AST/ALT/liver fat/liver fibrosis score/NAFLD fibrosis score	↑HDL
Scorletti et al. (synbiotics) (44)	UK	104	NAFLD	≥18	10 months	Polymerization	Maltodextrin	Weight/BMI/fat/FBS/HbA1c/TG/TC/LDL/HDL/ AST/ALT/GGT/MRS-measured liver/ELF score	↓NAFLD fibrosis score
Jafarvand et al. (48)	Iran	44	NAFLD	20–65	4 weeks	CoQ10	Placebo	BMI/WC/TG/TC/LDL/HDL/AST/ALT	↓WC/AST
Askari et al. (33)	Iran	50	NAFLD	20–66	12 weeks	Cinnamon	Wheat flour	FBS/QUICKI/HOMA/TC/TG/LDL/HDL/ASL/ALT/ GGT/hs-CRP	↓FBS/HOMA/T/LDL/TG/ASL/ALT/GGT/hs-CRP
Farsi et al. (49)	Iran	42	NAFLD	19–54	12 weeks	CoQ10	Starch	Weight/WC/HC/WHR/BMI/NAFLD/grade/TNF-α/adiponection/leptin/hs-CRP/AST/ALT/GGT/AST to ALT ratio	↓hs-CRP/AST/GGT/NAFLD grade/TNF-α/leptin; ↑adiponection
Izadi et al. (34)	Iran	70	NAFLD	20–55	8 weeks	Sour tea powder	Placebo	Weight/BMI/WC/TC/TG/LDL/HDL/AST/ALT	↓TG/ALT/AST/
Barchetta et al. (54)	Italy	65	NAFLD with DM	25–70	24 weeks	Cholecalciferol	Placebo	Hepatic fat fraction/BMI/WC/TC/TG/LDL/HDL/FBG/HbA1c/AST/ALT/r-GT/AST to ALT/FHOMA-IR/QUICKI/adiponectin	No difference
Bae et al. (35)	Korea	78	NAFLD with DM	20–70	12 weeks	Carnitine-orotate complex	Placebo	Weight/BMI/WC/FBS/HbA1c/HOMA-IR/HOMA-B/AST/ALT/γ-GT/TG/HDL/LDL/CT attenuation	↓ALT/HbA1c/liver attenuation values
Cansanção et al. (52)	Brazil	44	NAFLD	≥18	6 months	n-3 PUFA	Olive oil	ALT/AST/ALP/GGT/FBS/HbA1c/TG/TC/LDL/HDL/BMI/BMI/WC/WHR/liver fibrosis	↓ALP/liver fibrosis
Hosseinikia et al. (36)	Iran	90	NAFLD	18–65	12 weeks	Quercetin	Placebo	BMI/WHR/ALT/AST/GGT/TNF-α/hs-CRP/TC/TG/HDL/LDL	↓BMI/WHR/TNF-α
Farhangi et al. (50)	Iran	44	NAFLD	20–65	4 weeks	CoQ10	Placebo	FSG/insulin/HOMA-IR/QUICKI/AST/ALT/vaspin/chemerin/pentraxin	↓AST
Masoumeh et al. (55)	Iran	112	NAFLD	≥18	12 weeks	Whole grain foods	Usual cereals	Grade of fatty liver/ALT/AST/GGT/TG/TC/LDL/HDL/FBS/HOMA-IR/QUICKI/insulin	↓Grade of fatty/liver/ALT/AST/GGT
Darand et al. (21)	Iran	50	NAFLD	≥18	12 weeks	*Nigella sativa* seed powder	Starch	Weight/BMI/WC/HC/WHR/ALT/AST/GGT/hs-CRP/TNF-α/NF-κB/fibrosis grade/steatosis/percentage of steatosis	↓hs-CRP/TNF-α/NF-κb/percentage of steatosis
Mohamad Nor et al. (45)	Malaysia	46	NAFLD	≥18	6 months	Multi-strain probiotics	Placebo	Liver stiffness/AST/ALT/GGT/steatosis score/fibrosis score/BMI/TG/TC/fasting glucose	No difference
Mohammad Shahi et al. (37)	Iran	42	NAFLD	≥18	8 weeks	Phytosterol	Starch	AST/ALT/GGT/AST to ALT ratio/TC/TG/LDL/HDL/LDL to HDL ratio/VLDL/TC to HDL ratio/TNF-α/hs-CRP/adiponectin/leptin	↓AST/ALT/LDL
Rashidmayvan et al. (51)	Iran	44	NAFLD	20–60	8 weeks	NS oil	Paraffin oil	Weight/BMI/WC/WHR/FBS/TG/TC/HDL/LDL/VLDL/insulin/AST/ALT/GGT/TNF-α/hs-CRP	↓TG/TC/HDL/LDL/VLDL/FBS/AST/ALT/TNF-α/hs-CRP; ↑HDL
Zhang et al. (38)	China	74	NAFLD	25–65	12 weeks	Anthocyanin	Maltodextrin	Weight/BMI/WC/HC/WHR/AST/ALTNAFLD fibrosis score/TC/TG/LDL/DL/FBG/insulin/HOMA-IR/OGTT	↓ALT/HOMA-IR/2-h glucose
Chong et al. (47)	UK	42	NAFLD	25–70	10 weeks	VSL#3^®^ probiotic	Placebo	TC/TG/LDL/HDL/HbA1c//HOMA-IR/TAC/hs-CRP/ALT/AST/mode ASQ/NAFLD fibrosis risk score	No difference
Navekar et al. (39)	Iran	46	NAFLD with overweight/obesity	20–60	12 weeks	Turmeric powder	Placebo	FBS/insulin/HOMA-IR/leptin/AST/ALT	↓FBS/insulin/HOMA-IR/leptin
Ahn et al. (46)	Korea	68	NAFLD with overweight or obesity	19–75	12 weeks	Six bacterial species	Placebo	Weight/BMI/visceral fat/total fat mass/total body fat/visceral fat grade/WHR/CAP/liver stiffness/intra-hepatic fat fraction/TG/TC/glucose/insulin/AST/ALT/HOMA-I/total muscle mass/skeletal muscle/HDL/LPS/TNF-α	↓Intra-hepatic fat fraction/TG/weight
Cicero et al. (40)	Iran	65	NAFLD	≥18	8 weeks	Phospholipidated curcumin	Placebo	Weight/BMI/leptin/adiponectin/leptin:adiponectin/FBS/TG/TC/LDL/HDL/AST/ALT	↓leptin/leptin: adiponectin; ↑adiponectin/HDL
Namkhah et al. (41)	Iran	44	NAFLD with overweight/obesity	20–65	4 weeks	Naringenin	Placebo	NAFLD grades/NFS levels/weight/BMI/WC/AST/ALT/TG/TC/HDL/LDL	↓NAFLD grades/TG/TC/LDL; ↑HDL

NAFLD, non-alcoholic fatty liver disease; DHA, docosahexaenoic acid; EPA, eicosapentaenoic acid; PUFA, polyunsaturated fatty acid; SPB-201, powdered-water extract of Artemisia annua; DM, diabetes mellitus; BMI, body mass index; WC, weight circumference; WHR, waist to height ratio; FBS, fasting blood sugar; HOMA–IR, homeostasis model assessment for insulin resistance; QUICKI, quantitative insulin sensitivity check index; HDL-C, high-density lipoprotein cholesterol; LDL-C, low-density lipoprotein cholesterol; TC, total cholesterol; TG, triacylglyceride; ALT, alanine aminotransferase; AST, aspartate aminotransferase; GGT, γ-glutamyltransferase; ALP, alkaline phosphatase; CRP, C-reactive protein; TNF-α, tumor necrosis factor-α; UK, the United Kingdom. ↓, indicated a decrease in outcome measures. ↑, indicated an increase in outcome measures.

### 3.3. Risk of bias in studies

**Figure 2:**
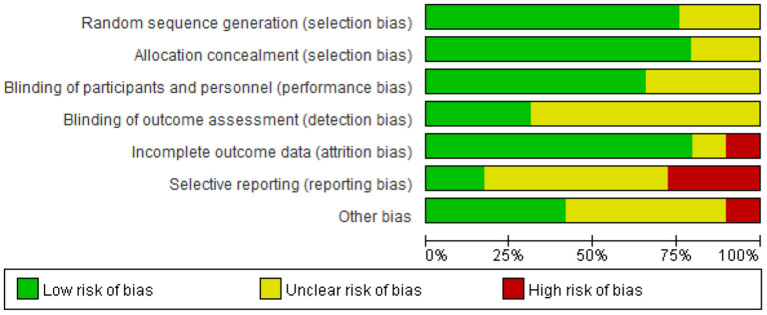
Risk of bias graph: review authors' judgements about each risk of bias item presented as percentages across all included studies.

**Figure 3:**
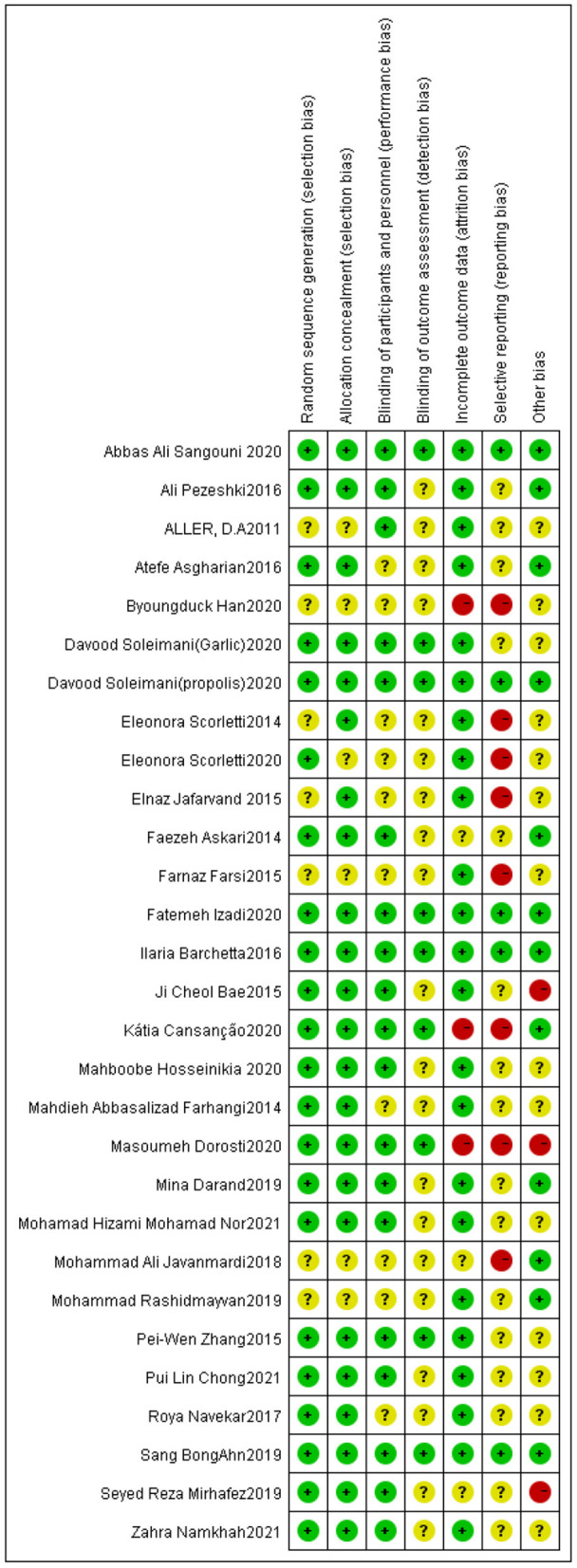
Risk of bias summary: review authors' judgements about each risk of bias item for each included study.

Out of all the studies, 21 trials (20, 21, 23, 30, 32–36, 38–41, 43, 45–47, 50, 52, 54, 55) had “good” quality, while five trials (42, 44, 48, 51, 53) were “fair”. Additionally, the remaining three trials (31, 37, 49) were “weak”. Figures 2, 3 show the results of each source of bias.

### 3.4. Effect of functional foods and dietary supplements on anthropometric parameters

**Figure 4:**
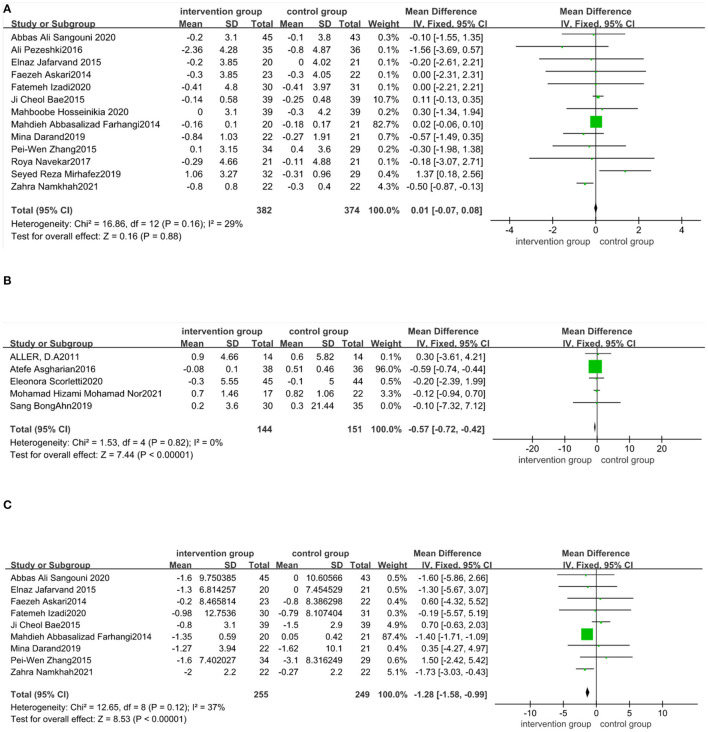
Forest plot of the effect of functional foods and dietary supplements on anthropometric indices (effect of **A**: antioxidants on BMI; **B**: probiotic/symbiotic/prebiotic on BMI; and **C**: antioxidants on WC).

The effect of functional foods and dietary supplements on BMI was examined in 22 clinical trials. It was seen that 13 out of 22 trials (21, 23, 30, 33–36, 38–41, 48, 50) were about antioxidants (phytonutrients and coenzyme Q10), five (42–46)on probiotic/symbiotic/prebiotic, three (51–53) on fatty acids, and one (54) on vitamin D. The meta-analysis showed no significant effect of antioxidants on BMI compared to the placebo (MD: 0.01 kg/m^2^; 95% CI: −0.07, 0.08, *P* > 0.05) (Figure 4A). There was no heterogeneity in these included studies (*I*^2^ = 29.0%, *P* = 0.16). As shown in Figure 4B, BMI significantly decreased (MD: −0.57 kg/m^2^; 95% CI: −0.72, −0.42, *P* < 0.05) after taking probiotic, symbiotic, and prebiotic in NAFLD patients, with no heterogeneity across studies (*I*^2^ = 0%, *P* = 0.82). Sensitivity analysis showed that the overall effect did not depend on any single study (Appendix S1 in the Supplementary material 1).

The proportion and type of fatty acids varied between the three studies; oil composition contained docosahexaenoic acid (DHA)/eicosapentaenoic acid (EPA)/linoleic acid/oleic acid and linolenic acid. Three studies used different oils as a placebo. All three studies showed that BMI did not change significantly in NAFLD patients after using fatty acids (51–53). Barchetta et al. (54) reported that BMI did not change after taking vitamin D and placebo.

Nine studies (21, 23, 33–35, 38, 41, 48, 50) reported the effect of supplements on WC. Our meta-analysis reported that WC declined significantly in nine antioxidant trials compared to the control group (MD: −1.28 cm; 95% CI: −1.58, −0.99, *P* < 0.05). The effect was homogeneous across the included trials (*I*^2^ = 37%, *P* = 0.12) (Figure 4C). The effects of probiotic/symbiotic/prebiotic on WC have not been reported. Sensitivity analysis showed that the exclusion of any trials did not affect the results (Appendix S2 in the Supplementary material 1). Four studies (51–54) noted no changes in the WC after taking fatty acid (three trials) and vitamin D (one trial).

No publication bias was found for BMI (Begg's test *P* = 0.82; Egger's test *P* = 0.600) and WC (Begg's test *P* = 0.348; Egger's test *P* = 0.829).

### 3.5. Effect of functional foods and dietary supplements on the liver function

Twenty-nine datasets evaluated the effect of functional foods and dietary supplements on ALT. Eighteen out of 29 trials were about antioxidants (phytonutrients and coenzyme Q10), six (42–47) on probiotic/symbiotic/prebiotic, three (51–53) on fatty acids, one (55) on whole grain, and one (54) on vitamin D.

**Figure 5:**
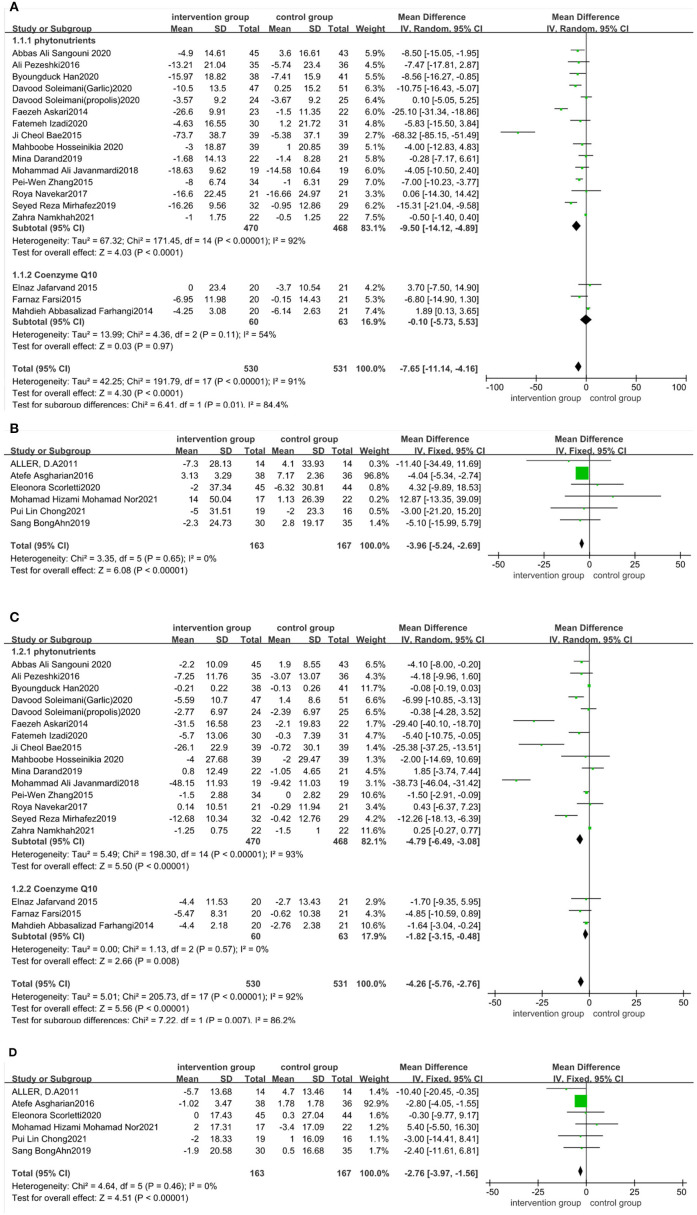
Forest plot of the effect of functional foods and dietary supplements on liver function (effect of **A**: antioxidants on ALT; **B**: probiotic/symbiotic/prebiotic on ALT; **C**: antioxidants on AST; and **D**: probiotic/symbiotic/prebiotic on AST).

There was a statistical significance in the mean difference of ALT levels in the antioxidant-supplemented group compared to the control group (MD: −7.65 IU/L; 95% CI: −11.14, −4.16, *P* < 0.0001). There was high heterogeneity across studies (*I*^2^ = 91%, *P* < 0.00001). Subgroup analysis based on the type of antioxidants (phytonutrients or coenzyme Q10) was performed using data from 18 studies. There was statistically significant difference in ALT reductions between phytonutrients group and control group (MD: −9.5 IU/L; 95% CI: −14.12, −4.89, *P* < 0.0001), but high heterogeneity of these studies still existed (*I*^2^ = 92%, *P* < 0.00001). Heterogeneity was attenuated among those studies taking coenzyme Q10 (*I*^2^ = 54%, *P* = 0.11) (Figure 5A), but the ALT reductions had no difference. The intake of probiotic, symbiotic, and prebiotic supplements led to a more significant decrease in ALT compared with control (MD: −3.96 IU/L; 95% CI: −5.24, −2.69, *P* < 0.001), with no heterogeneity across studies (*I*^2^ = 0%, *P* = 0.65) (Figure 5B). Sensitivity analysis indicated that no trial changed the pooled effect size (Appendix S3 in the Supplementary material 1). Publication bias were seen for ALT (Begg's test *P* = 0.722; Egger's test *P* = 0.016).

The effect of functional foods and dietary supplements on AST levels was examined in 29 clinical trials. The literature distribution on AST was consistent with that on ALT. Our meta-analysis revealed a significant reduction in AST levels after taking antioxidant supplements (MD: −4.26 IU/L; 95% CI: −5.76, −2.76, *P* < 0.00001), with substantial heterogeneity (*I*^2^ = 92%, *P* < 0.00001). Studies of phytonutrients or coenzyme Q10 were subgroup analyzed. The AST reductions were significantly different between the phytonutrients group and control group (MD: −4.79 IU/L; 95% CI: −6.49, −3.08, *P* < 0.00001), with high heterogeneity among those studies (*I*^2^ = 93%, *P* < 0.00001). Meanwhile, the AST reductions had significant difference in studies taking coenzyme Q10 (MD: −1.82 IU/L; 95% CI: −3.15, −0.48, *P* = 0.008), with no heterogeneity (*I*^2^ = 0%, *P* = 0.57) (Figure 5C). The AST levels after taking probiotic, symbiotic, and prebiotic showed a decrease in the antioxidant-supplemented group compared to the control group (MD: −2.76; 95% CI, −3.97, −1.56, *P* < 0.0001), with no heterogeneity across studies (*I*^2^ = 0%, *P* = 0.46) (Figure 5D). Sensitivity analysis revealed that no specific study affected pooled effects (Appendix S4 in the Supplementary material 1). publication bias was seen for AST levels (Begg's test *P* = 0.985; Egger's test *P* = 0.000).

In three trials (51–53) on fatty acids, Rashidmayvan et al. (51) reported that *Nigella sativa* oil containing linoleic acid, oleic acid, and linolenic acid reduced ALT and AST levels, whereas Cansanção et al. (52) and Scorletti et al. (53) found that supplements containing DHA and EPA did not affect ALT and AST levels. Barchetta et al. (54) did not observe any discrepancy in AST and ALT levels between the vitamin D and control groups. Masoumeh et al. (55) found that ALT and AST levels decreased significantly after taking whole grain compared to the control group. The outcome indicators of hepatic fibrosis and steatosis were too few to be described.

### 3.6. Effect of functional foods and dietary supplements on lipid profiles

**Figure 6:**
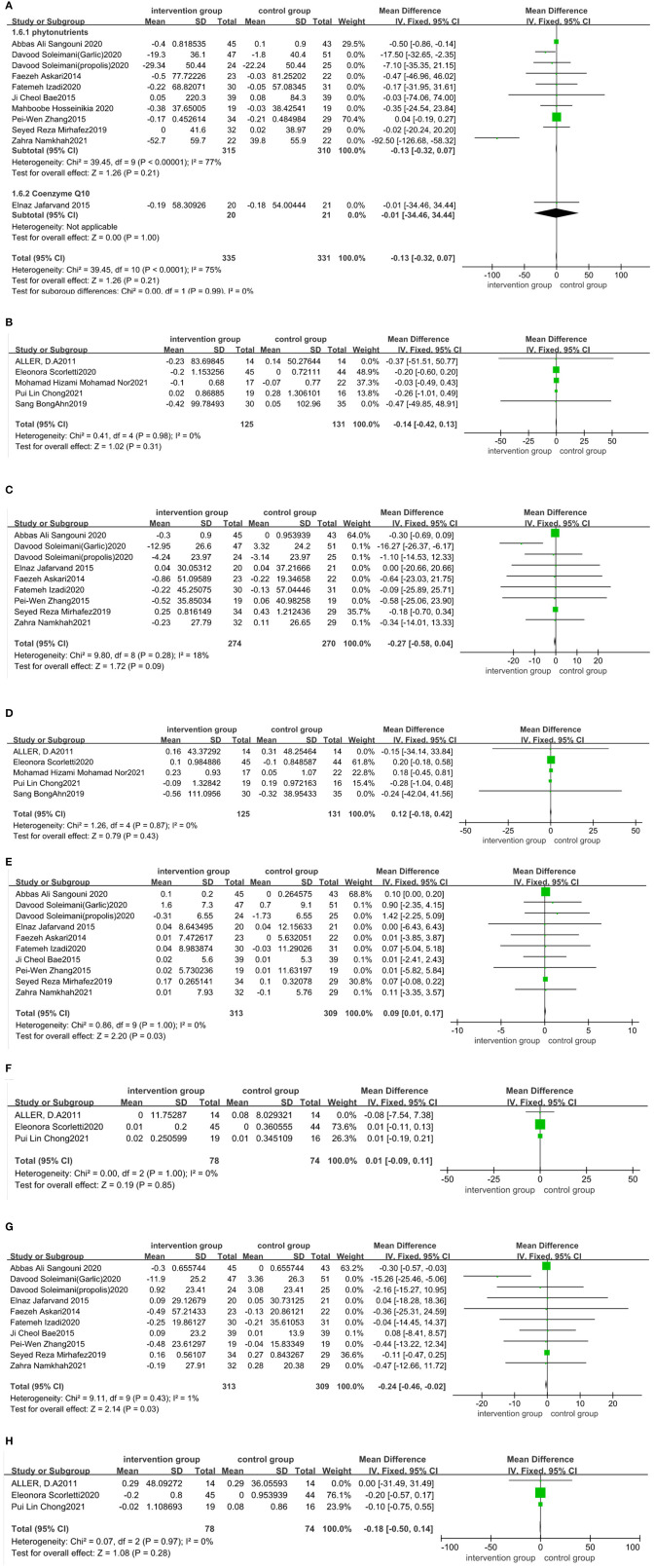
Forest plot of the effect of functional foods and dietary supplements on lipid profiles (effect of **A**: antioxidants on TG; **B**: probiotic/ symbiotic/prebiotic on TG; **C**: antioxidants on TC; **D**: probiotic/symbiotic/prebiotic on TC; **E**: antioxidants on HDL-C; **F**: probiotics/symbiotic/prebiotic on HDL-C; **G**: antioxidants on LDL-C; and **H**: probiotic/symbiotic/prebiotic on LDL-C).

Eighteen trials (20, 23, 32–36, 38, 40–42, 44–48, 51, 53–55) assessed the effect of functional foods and dietary supplements on TG. The quantitative analysis of TG values indicated no significant reduction in TG after antioxidant supplementation [11 trials (20, 23, 32–36, 38, 40, 44, 48)] (MD: −0.13 mg/dL; 95% CI: −0.32, 0.07, *P* > 0.05) with high heterogeneity (*I*^2^ = 75%, *P* < 0.05) (Figure 6A). We were not able to perform a subgroup analysis to assess the source of heterogeneity as the number of papers on coenzyme Q10 subgroup was small. Our meta-analysis of five trials (43–47) involving probiotic, symbiotic, and prebiotic intake illustrated that these supplements had an unnoticeable effect on TG reduction (MD: −0.14 mg/dL; 95% CI: −0.42, 0.13, *P* > 0.05), with no heterogeneity (*I*^2^ = 0%, *P* > 0.05) (Figure 6B). Sensitivity analysis showed that the results did not depend on any single study (Appendix S5 in the Supplementary material 1).

Consistently, nine datasets (30, 32–35, 39, 41, 42, 49) indicated no significant reduction in TC levels after taking antioxidant supplements compared to the control group (MD: −0.27 mg/dL; 95% CI: −0.58, 0.04, *P* > 0.05) (Figure 6C), with no heterogeneity (*I*^2^ = 18%, *P* > 0.05). Five datasets (43–47) on probiotic, symbiotic, and prebiotic supplements reported no significant decrease in TC levels after intervention (MD: 0.12 mg/dL; 95% CI: −0.18, 0.42, *P* > 0.05) (Figure 6D), with no heterogeneity (*I*^2^ = 0%, *P* > 0.05). The results of the sensitivity analysis showed that excluding either study from the analysis did not change the overall effect (Appendix S6 in the Supplementary material 1).

The pooled effect size of 10 trials (30, 32–36, 39, 41, 42, 49) reported a significant impact of the antioxidant supplements on HDL-C (MD: 0.09 mg/dL; 95% CI: 0.01, 0.17, *P* < 0.05) and LDL-C (MD: −0.24 mg/dL; 95% CI: −0.46, −0.02, *P* < 0.05), with no heterogeneity (*I*^2^ = 0%, *P* > 0.05; *I*^2^ = 1%, *P* > 0.05, respectively) (Figures 6E, G). Three trials (43–45) reported no significant effect of probiotic, symbiotic, and prebiotic supplementation on HDL-C (MD: 0.01 mg/dL; 95% CI: −0.09, 0.11, *P* > 0.05) and LDL-C (MD: −0.18 mg/dL; 95% CI: −0.5, 0.14, *P* > 0.05), with no heterogeneity (*I*^2^ = 0%, *P* > 0.05 for both) (Figures 6F, H). We performed a sensitivity analysis and found that excluding a particular study from the analysis did not change the overall effect on HDL-C and LDL-C (Appendices S7, S8 in the Supplementary material 1).

Four trials (52–55) reported no difference in TG, TC, HDL-C, and LDL-C levels of DHA/EPA, vitamin D, or whole grainarms of the trial. Rashidmayvan et al. (51) reported that *Nigella sativa* oil decreased TG, TC, HDL-C, and LDL-C levels with significant differences between the NS seed and placebo groups.

No evidence of publication bias was seen for TG (Begg's test *P* = 0.753; Egger's test *P* = 0.120), TC (Begg's test *P* = 0.488; Egger's test *P* = 0.424), LDL-C (Begg's test *P* = 0.913; Egger's test *P* = 0.0.138), and HDL-C (Begg's test *P* = 0.743; Egger's test *P* = 0.084).

## 4. Discussion

NAFLD is considered a metabolic disorder that is closely related to lifestyle. The number of studies about the relationship between diet, nutrition, food, and NAFLD has increased in recent years (56), but there have been no recommendations in the guidelines in terms of this relationship. This systematic review and meta-analysis summarized the adjuvant therapy effects of various nutritional interventions on NAFLD, including antioxidants, probiotic/symbiotic/prebiotic, fatty acid supplements, vitamin D, and whole grain, providing guidance for clinical application.

A total of 1,907 patients from 29 trials were included in this review. Eighteen trials assessed the impact of antioxidants on NAFLD. The mechanism may be related to their antioxidant activity and the ability to scavenge free radicals, such as inhibiting the early formation of lipid peroxides in the liver, blocking the transmission process of free radicals by interrupting the chain reaction, or indirectly scavenging free radicals by acting on enzymes related to free radicals (57, 58). For humans, it can reduce liver inflammation, decrease oxidative stress, inhibit lipid oxidation in serum, and finally reduce serum aminotransferase and improve serum lipids (24). This meta-analysis found that the antioxidants significantly reduced WC, ALT, AST, and LDL-C and increased HDL-C in NAFLD patients but did not change the BMI, TC, and TG levels. This meta-analysis found that the antioxidants significantly reduced WC, ALT, AST, and LDL-C and increased HDL-C in NAFLD patients but did not change the BMI, TC, and TG levels. The positive results were consistent with the action mechanism of antioxidants, whose hypolipidemic properties were related to the reduction of liver adipogenesis (20). With the action of antioxidants, inflammation induced by hepatocyte steatosis was improved, and ALT/AST levels were significantly decreased. WC, which is closely related to visceral fat deposition (59), also decreased significantly. Moreover, the amount of cholesterol decreased, especially oxidized LDL, HDL increased (60), which was consistent with the results of this study. On the other hand, TC reflects the sum of all LDL-C, HDL-C and VLDL in serum. In this study, LDL-C decreased and HDL-C increased, so the resulting overall lack of change can be understood. The lack of significant changes in TG may be due to the fact that only a small part of TG is synthesized by the body itself, and most TG is obtained from diet, which is influenced by many factors (61). Finally, elevated BMI is not a prerequisite for the diagnosis of NAFLD, and many of the included studies included patients with normal BMI, so it is not difficult to understand the lack of changes in BMI levels caused by antioxidants. However, due to the different dosages and types of antioxidants used in each trial, its effect should be further investigated in clinical application.

Six randomized trials indicated that taking probiotic/symbiotic/prebiotic decreased BMI, ALT, and AST levels but did not have favorable effects on blood lipid levels compared to the control group. The results were inconsistent with the previous review (62). This difference might be related to the type of strains. Studies have shown that different strains have different effects on liver function and blood lipid levels (12, 15, 63). However, since few trials were included in the study, the respective effects of probiotic/symbiotic/prebiotic could not be compared through subgroup analysis. In general, their adjuvant therapeutic effect on NAFLD is clear. The potential mechanism may involve regulating human metabolism by decreasing inflammatory markers and altering lipid profile (3). However, the dose and duration of their clinical application are ill-defined; therefore, more multi-central clinical research is needed to provide detailed guidance in the future.

Three RCTs reported the role of fatty acid supplementation in the treatment of NAFLD, but the results showed significant heterogeneity, which might be related to the dosage and durations of the trials. The dosages ranged from 1,000 to 4,000 mg, and the durations were from 8 to 24 weeks (51–53), which was in agreement with the result of another review (64). Therefore, additional trials are needed to demonstrate the effectiveness of fatty acids in NAFLD patients.

The effect of vitamin D may be related to reduced inflammatory markers (65), while whole grain could change intestinal microbial metabolites (66, 67). There were only a few articles on vitamin D and whole grain in this study because several interventions combined physical exercise with other supplements had to be excluded. At present, vitamin D combined with other treatments has been proven as an adjunctive therapy to improve liver function for NAFLD patients (68, 69). However, the articles on whole grain were scarce; therefore, larger sample size studies will be needed to provide evidence for the use of the low-cost whole grain.

This review has several limitations. Although our study showed that functional food and dietary supplements improved AST and ALT in patients with NAFLD, the source of all heterogeneity could not be explained by the subgroup analysis, especially most of the studies did not identify the stage of NAFLD and the included subjects in some studies were from a single ethnicity- mainly from Asia. Moreover, functional foods and dietary supplements used in these articles were produced in different countries with various dosages, which might have an impact on the results. Multi-center clinical research from different countries should be carried out to explore effective doses using uniform sources of functional foods and dietary supplements in the clinic. In addition, we only searched studies published in English in databases such as PubMed, ISI Web of Science, Cochrane library, and Embase. Thus, additional relevant studies might exist, and additional reviews may be required.

## 5. Conclusion

The current study suggests that antioxidant and probiotic/symbiotic/prebiotic supplements alone or in combination with other therapies may be a promising regimen for NAFLD patients. However, the usage of fatty acids, vitamin D, and whole grain in clinical treatment is uncertain. Further exploration of the efficacy ranks of functional foods and dietary supplements is needed to provide a reliable basis for clinical application.

## Data availability statement

The original contributions presented in the study are included in the article/Supplementary material, further inquiries can be directed to the corresponding authors.

## Author contributions

H-hY and L-lW conceived, designed the study, searched databases, screened articles, extracted data, and performed statistical analyses. P-hZ contributed to the revision of the manuscript. All authors contributed to the writing and revision of the manuscript.
