# Supplementary material for: Functional foods and dietary supplements in the management of non-alcoholic fatty liver disease: A systematic review and meta-analysis
Source: Front Nutr. 2023 Feb 14;10:1014010. doi: 10.3389/fnut.2023.1014010 (PMC9971819; doi:10.3389/fnut.2023.1014010)
Supplement: Supplementary file 1 [file Data_Sheet_1.doc]

Supplementary materials 1


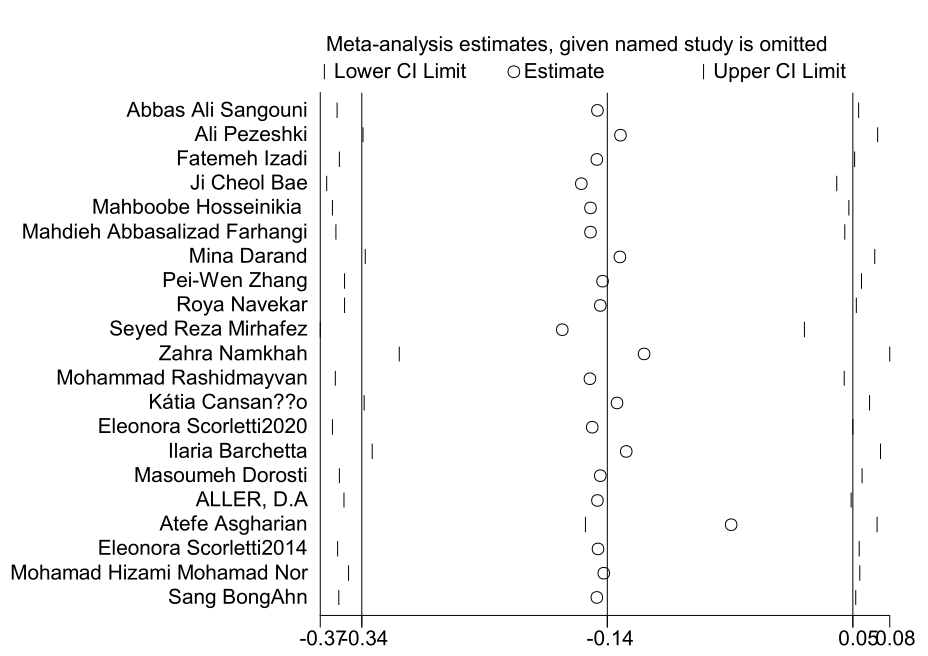


Appendix S1 Sensitivity analysis for BMI


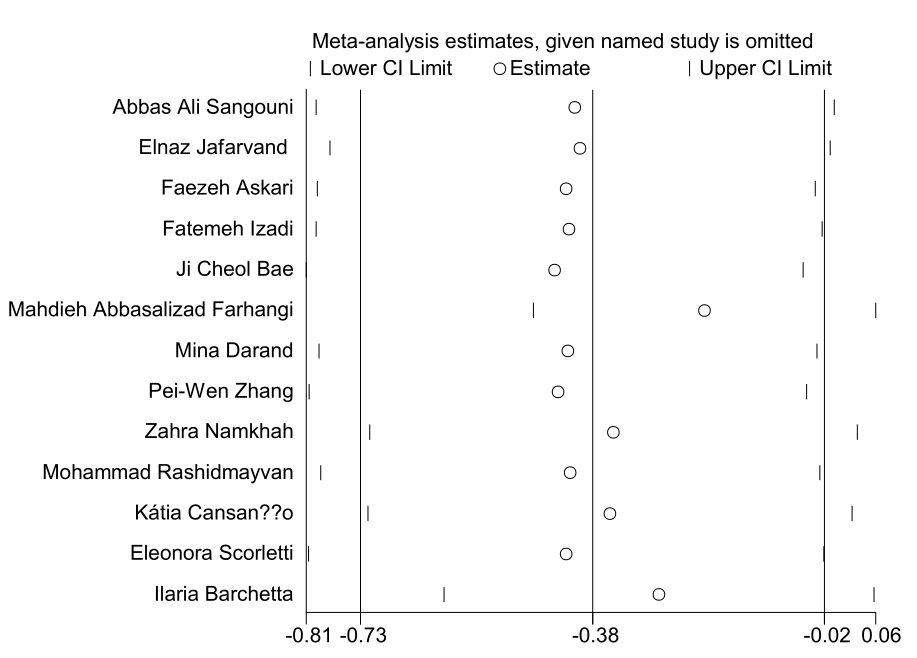


Appendix S2 Sensitivity analysis for WC


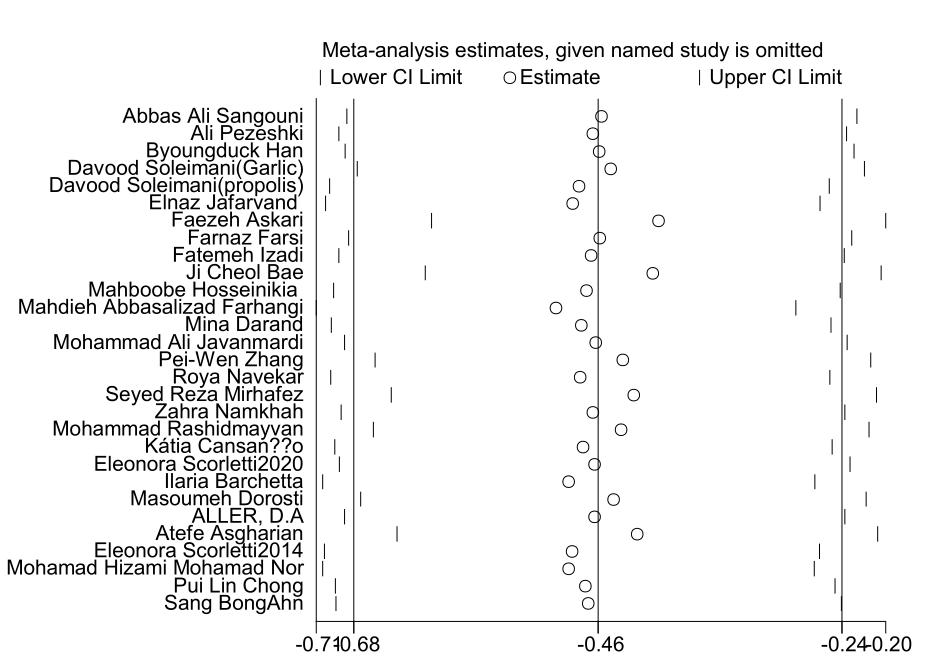


Appendix S3 Sensitivity analysis for ALT


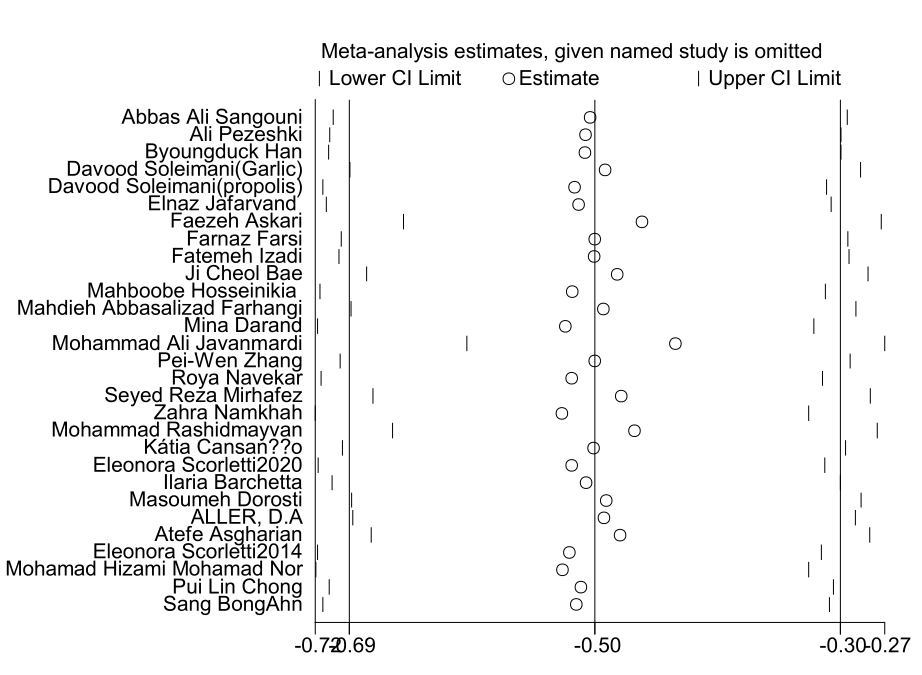


Appendix S4 Sensitivity analysis for AST


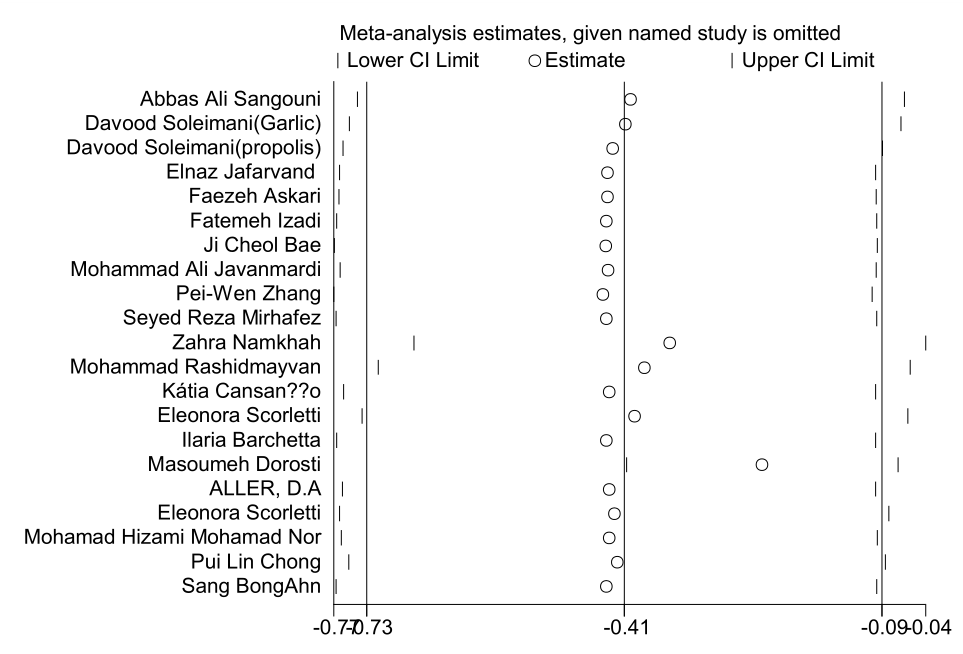


Appendix S5 Sensitivity analysis for TG


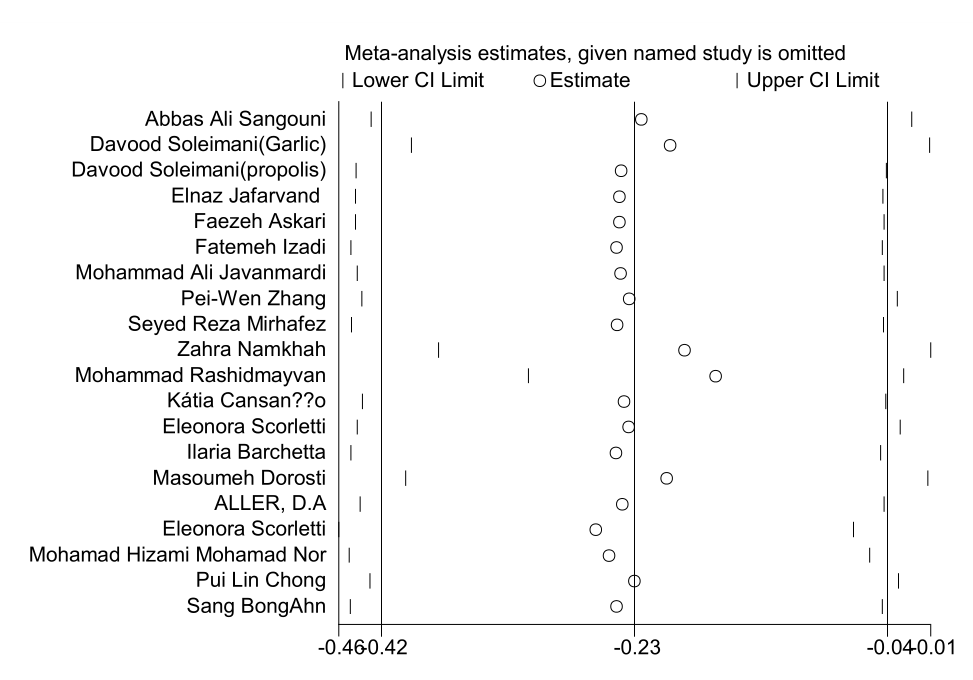


Appendix S6 Sensitivity analysis for TC


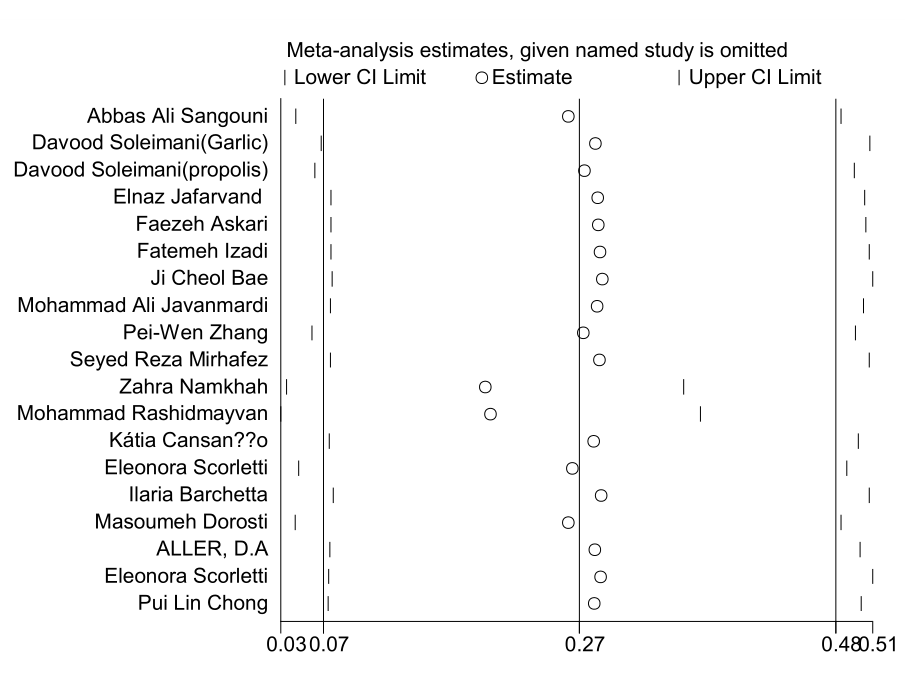


Appendix S7 Sensitivity analysis for HDL-C


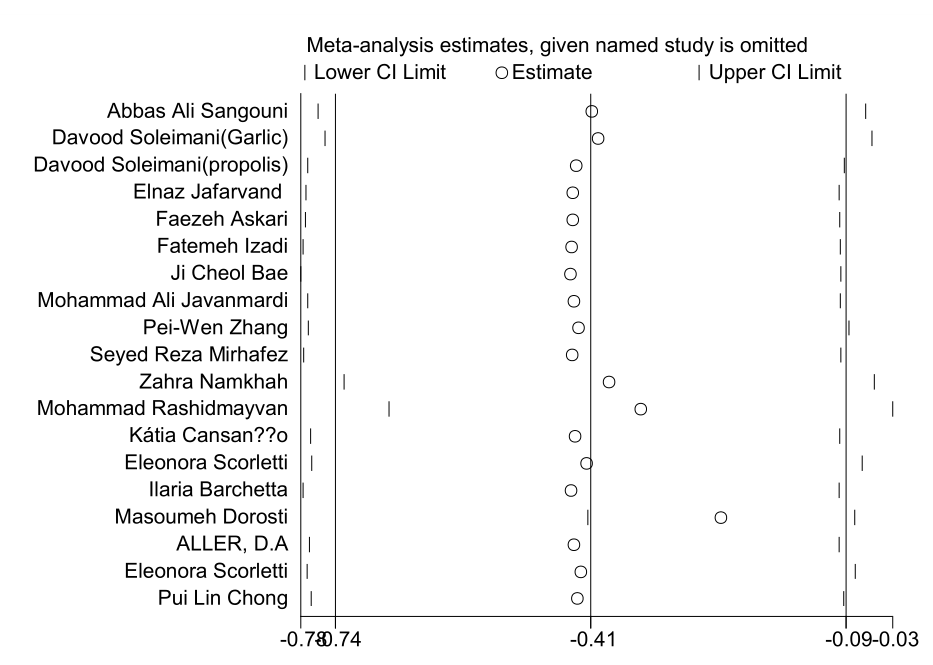


Appendix S8 Sensitivity analysis for LDL-C
