# Supplementary material for: Functional foods and dietary supplements in the management of non-alcoholic fatty liver disease: A systematic review and meta-analysis
Source: Front Nutr. 2023 Feb 14;10:1014010. doi: 10.3389/fnut.2023.1014010 (PMC9971819; doi:10.3389/fnut.2023.1014010)
Supplement: Supplementary file 2 [file Data_Sheet_2.doc]

Supplementary materials 2


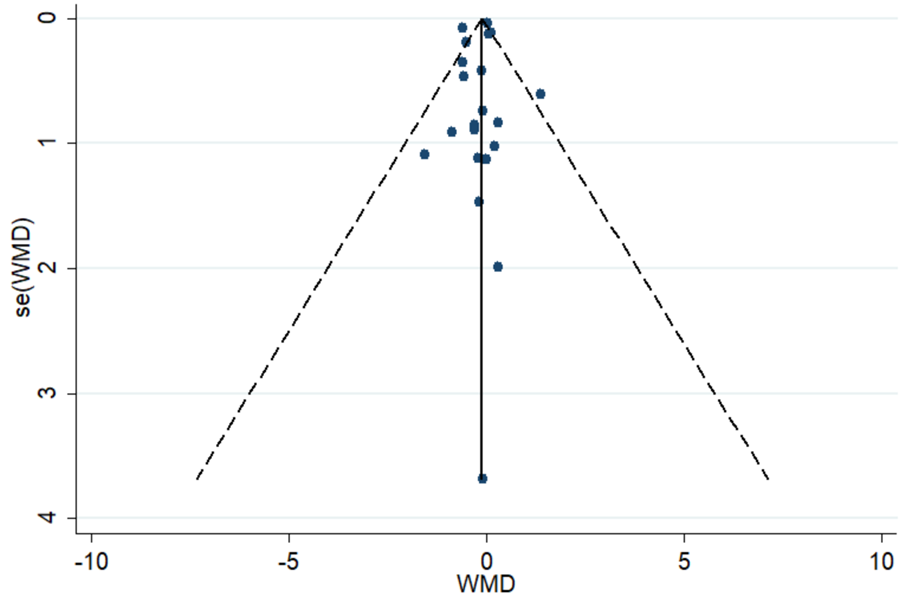


Appendix S1 funnel plots with pseudo 95% CIs for BMI


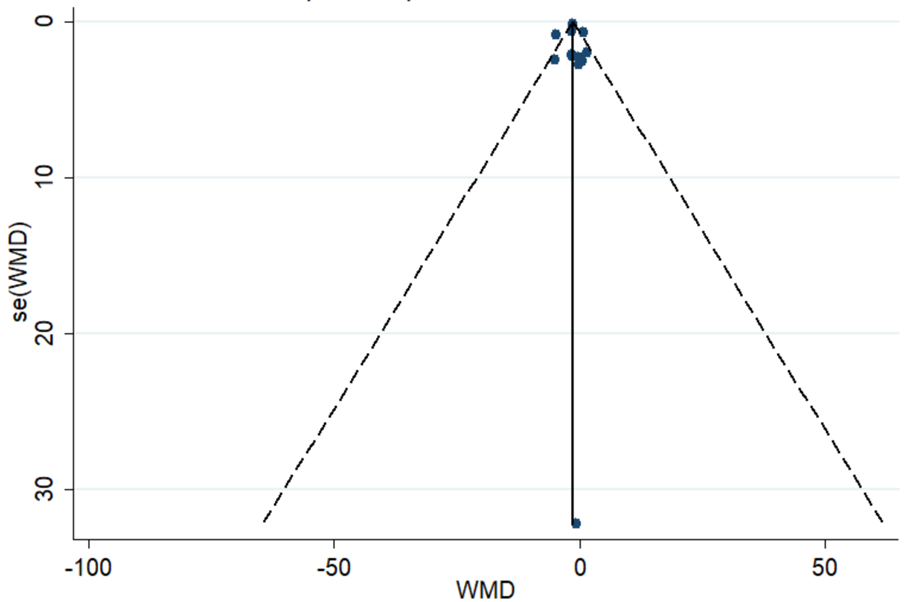


Appendix S2 funnel plots with pseudo 95% CIs for WC


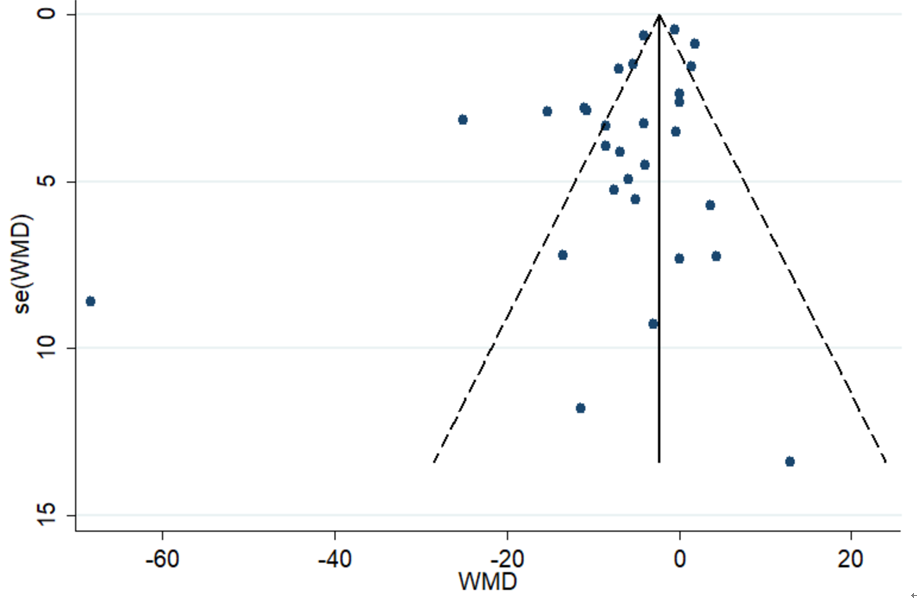


Appendix S3 funnel plots with pseudo 95% CIs for ALT


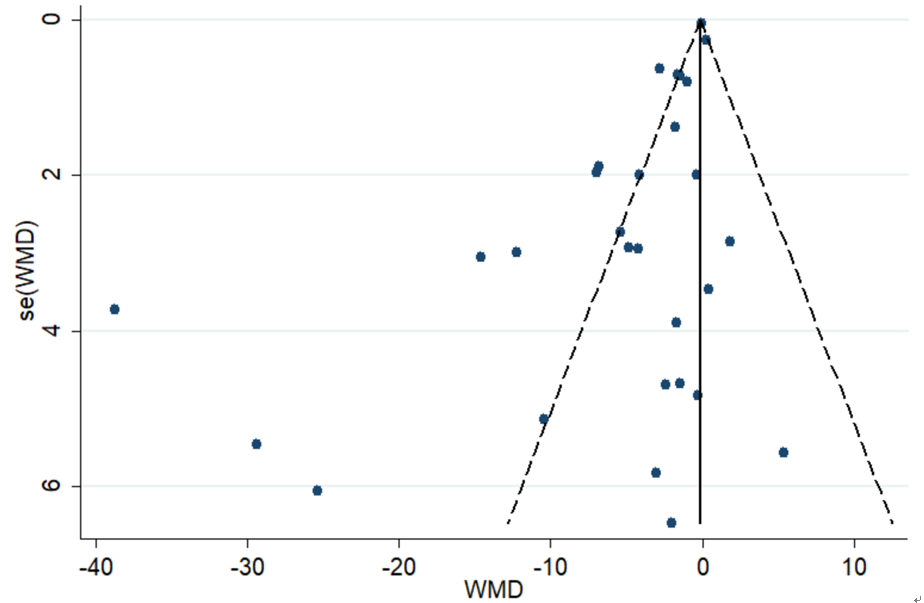


Appendix S4 funnel plots with pseudo 95% CIs for AST


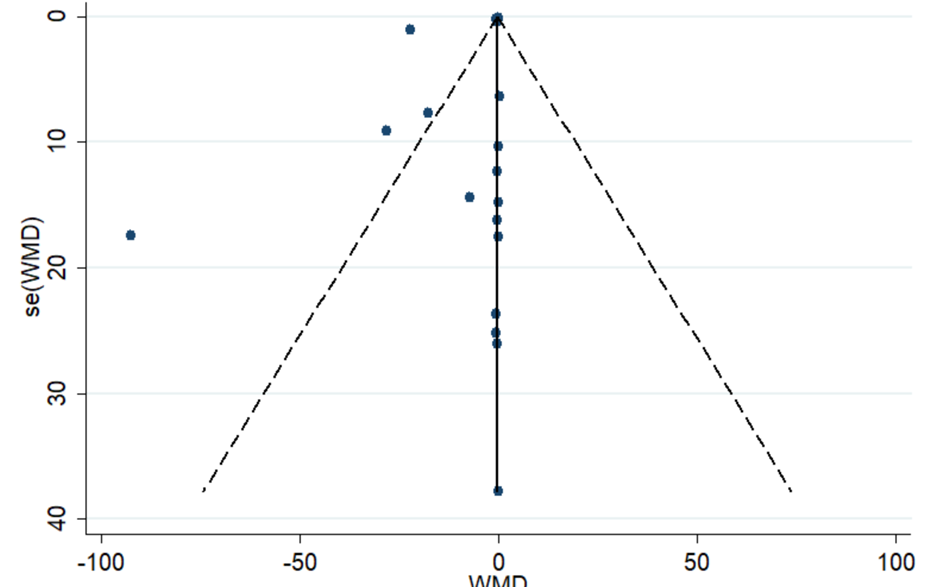


Appendix S5 funnel plots with pseudo 95% CIs for TG


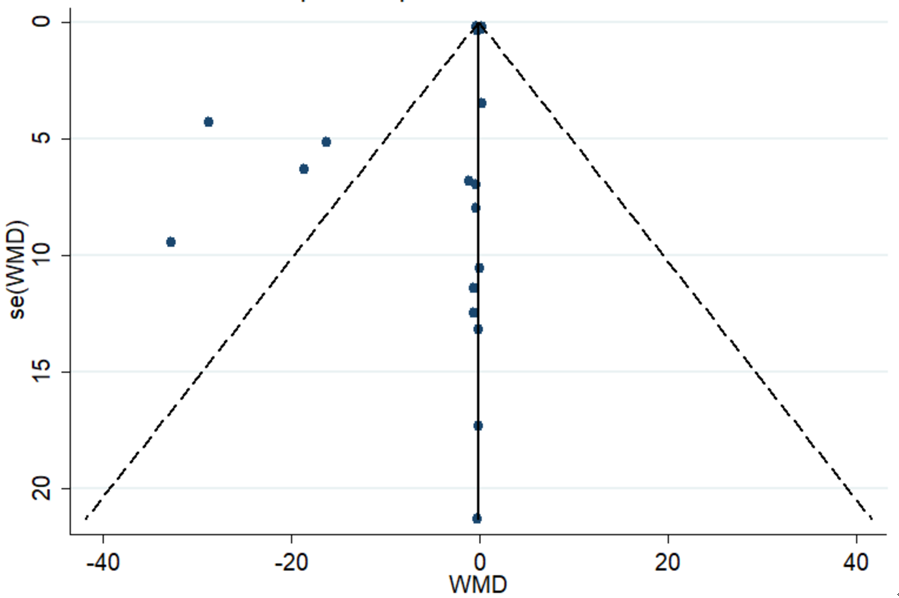


Appendix S6 funnel plots with pseudo 95% CIs for TC


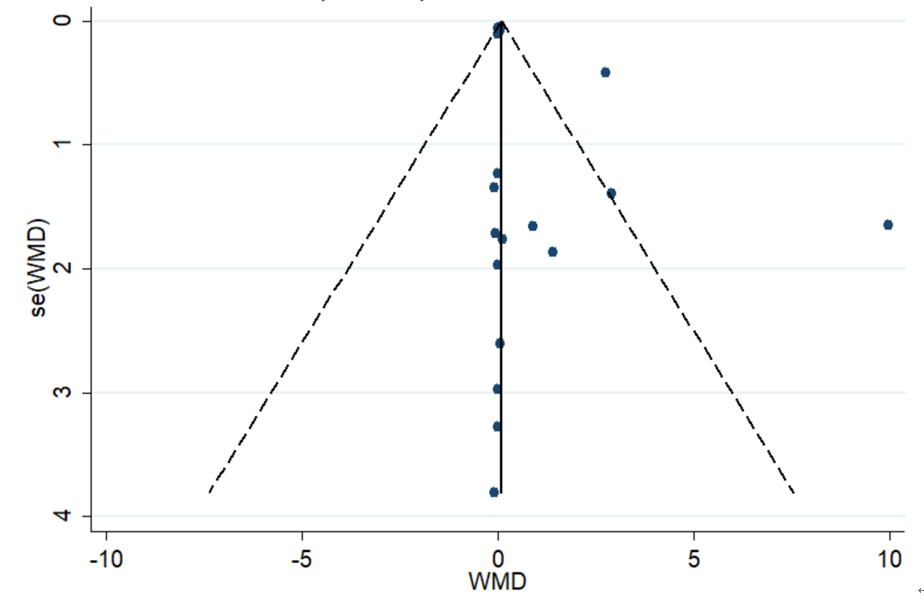


Appendix S7 funnel plots with pseudo 95% CIs for HDL-C


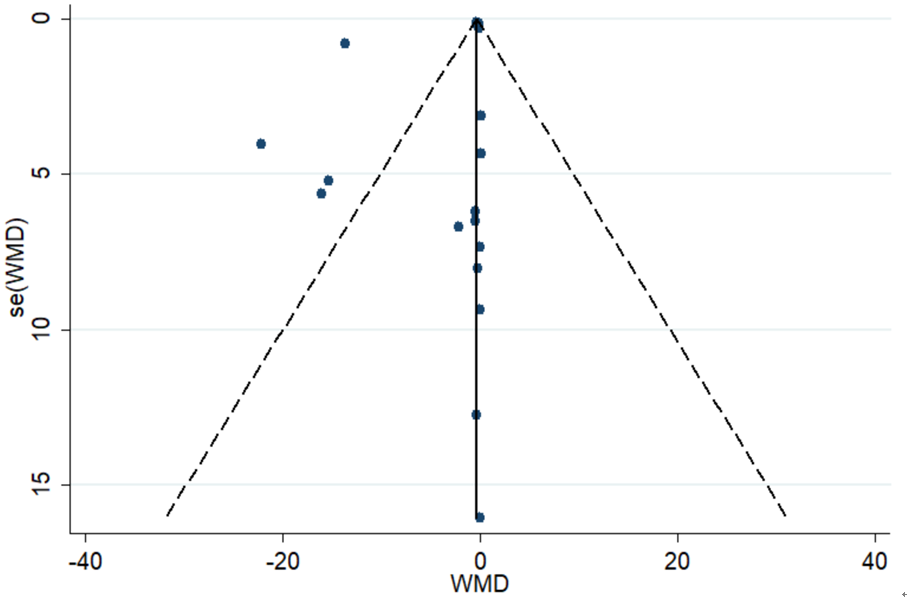


Appendix S8 funnel plots with pseudo 95% CIs for LDL-C
